# A super-family of genes coding for secreted salivary gland proteins from the Hessian fly, *Mayetiola destructor*

**DOI:** 10.1673/2006.06.12.1

**Published:** 2006-08-03

**Authors:** Ming-Shun Chen, John P. Fellers, Yu Cheng Zhu, Jeffrey J. Stuart, Scot Hulbert, Mustapha El-Bouhssini, Xiang Liu

**Affiliations:** 1 USDA-ARS, Plant Science and Entomology Research Unit, 4007 Throckmorton Hall, Manhattan, KS 66506; 2 Department of Entomology, 123 Waters Hall, Kansas State University, Manhattan, KS 66506; 3 Department of Plant Pathology, 4027 Throckmorton Hall, Kansas State University, Manhattan, KS 66506; 4 USDA-ARS-JWDSRC, PO Box 346/141 Exp Stn Rd, Stoneville, MS 38776; 5 Department of Entomology, Purdue University, West Lafayette, Indiana 47907; 6 International Center for Agricultural Research in the Dry Areas, Aleppo, Syria

**Keywords:** gene superfamily, gene cluster, SSGP secreted salivary gland protein

## Abstract

We have previously characterized a gene coding for the secreted-salivary-gland-protein 11A1 (SSGP-11A1) from the Hessian fly,Mayetiola destructor (Say) (Diptera Cecidomyiidae). Here we report the cloning and characterization of three new genes coding for proteins designated SSGP-11B1, SSGP-11C1, and SSGP-11C2, and their relationship with the SSGP-11A1-encoding gene. Based on their structural conservation, similar regulation, and clustered genomic organization, we conclude that the four genes represent a gene superfamily, designated *SSGP-11*, which originated from a common ancestor. Cloning, Southern blot and *in situ* hybridization data suggest that each of the *SSGP-11* families has multiple members that cluster within short chromosome regions. The presence of a secretion signal peptide, the exclusive expression in the larval stage, and the clustered genomic organization indicate that this superfamily might be important for Hessian fly virulence/avirulence.

## INTRODUCTION

The Hessian fly, Mayetiola destructor (Say) (Diptera Cecidomyiidae), is one of the most destructive pests of wheat in Southern Europe, Northern Africa, Central Asia, and North America (Hatchett et al. 1987). Resistance genes in wheat have long been used for controlling this insect (Ratcliff and Hatchett 1997). Host-plant resistance provides an effective, cost-efficient, and environment-friendly way to control many important insect pests (Gracen 1986; Ratcliffe and Hatchett 1997). The challenge for the host-plant resistance strategy is the constant development of new biotypes that can overcome the resistance of deployed genes (Ratcliffe et al. 1994; Ratcliffe et al. 2000). To improve the durability of host-plant resistance, we need to understand how new biotypes arise. At present, little is known about the genetic basis for insect biotype differentiation. Thirty-one resistance genes have been identified and many biotypes have been isolated and are being maintained in laboratory collections of the Hessian fly (Ratcliff and Hatchett 1997; Martin-Sanchez et al. 2003; Williams et al. 2003). The availability of a large collection of host resistance genes and insect biotypes for this insect provides an ideal model system to reveal the genetic basis for biotype differentiation.

Numerous effector proteins from bacterial pathogens have been characterized (Leach et al. 1996; Dent et al. 1997). There is considerable evidence that these effector proteins are essential for microbes to be successful (Bai et al. 2000). Effector proteins that are recognized by specific plant-resistance-gene products and, thus, cause avirulence are designated *Avr* proteins (Baker et al. 1997). Pathogenic fungi also secret *Avr* proteins into plant tissues, but less is known about this process (Orbach et al. 2000).

The Hessian fly interacts with wheat in a typical gene-for-gene specificity (Hatchett and Gallun 1970; Rider et al. 2002). Like pathogens, Hessian fly larvae apparently inject substances into host plants *via* their salivary glands during feeding (Byers and Gallun 1971;Hatchett et al 1990). Thus the genetic determinants for Hessian fly biotypes could be those genes that encode secreted salivary gland proteins (SSGP) that are injected into host plants. The injected substances could be determinants for Hessian fly virulence and the variations in these substances could be determinants for biotype differentiation. As the first step in determining the relationship between SSGP and virulence/avirulence of specific Hessian fly biotypes, we systematically analyzed the genes coding for SSGP from Hessian fly larvae following an expressed sequence tag (EST) approach. Numerous SSGP-encoding genes have been identified as a result of this analysis (Chen et al. 2004; Liu et al. 2004; Chen et al., unpublished). We previously characterized a gene coding for a small (7.1 kDa) protein designated SSGP-11A1 (Liu et al. 2004). Phylogenetic analysis of SSGP-11A1 together with other putative SSGP revealed that there are three other groups of SSGP that belong to the same sublineage group. The three new groups were named SSGP-11B1, SSGP-11C1, and SSGP-11C2, respectively. Here we report the isolation and characterization of the corresponding genes for these three new groups and their evolutionary relationship with the SSGP-11A1-encoding gene.

## MATERIALS AND METHODS

### Hessian flies

Insects were from a laboratory colony that originated from insects collected in Ellis County, Kansas (Gagne and Hatchett 1989). Since then, the insects were maintained on susceptible wheat seedlings in environmental chambers at 20° C and 12:12 (L:D) photoperiod. The majority of the insects were biotype GP (Great Plains) although biotypes A, B and others were also found in low frequencies (Harris and Rose 1989).

### Library construction, screening, and sequencing

cDNA and BAC library construction, library screening and sequencing were conducted as described previously (Chen et al. 2004; Liu et al. 2004). The nucleotide sequences of genes and cDNAs from this article have been deposited in GenBank under accession nos. AY828552 to AY828563.

### RNA isolation and Northern blot analysis

Total RNA was extracted from salivary glands or whole insects using TRI reagent^TM^ (Molecular Research Inc., www.mrcgene.com) following the protocol provided by the manufacturer. For Northern blots, equal amounts (5 μg) of total RNA were separated on a 1.2% agarose gel containing formaldehyde and blotted onto GeneScreen membrane (Perkin Elmer,www.perkinelmer.com). The membrane was incubated at 80° C for two hours. Hybridization and washing conditions are the same as described elsewhere (Liu et al. 2004). For dot blot analysis, 2.5 μg of total RNA was used for each sample.

### Sequence analysis

Open-Reading-Frame (ORF) and sequence similarity analysis were performed using ORF finder and various BLAST programs on the website (**http://www.ncbi.nlm.nih.gov/**) of the National Center for Biotechnology Information (Bethesda, MD). Analysis for secretion signal peptides was carried out using SignalP (Center for Biological Sequence Analysis, Technical University of Denmark, **http://www.cbs.dtu.dk/services/SignalP/**) and PSORT II analysis (Prediction of Protein Sorting Signals and Localization Sites in Amino Acid Sequences, **http://psort.nibb.ac.jp/**). Molecular weight calculations and pI prediction of mature proteins were carried out using the ‘Compute pI/Mw tool’ **(http://us.expasy.org/tools/pi_tool.html**).

### Fluorescent *in situ* hybridization

Polytene chromosomes were isolated from the salivary glands of second instar larvae and prepared as described by Shukle and Stuart (1995). Probes were prepared by labeling DNA (1 μg) with digoxigenin-conjugate dUTP by nick translation. Hybridizations were performed with 40–100 ng of denatured probe DNA on each chromosome preparation in 10 μl of hybridization solution (10% dextran sulfate, 2x SSC, 50% formaldehyde, and 20 μg of herring sperm DNA) at 37° C for 12 hours. Detection was performed using rhodamine-conjugated anti-digoxigenin and Alexa Fluor 488-conjugated strepavidin. Chromosomes were counterstained with DAPI. Digital images were taken with UV optics using an ORCA-ER (Hamamatsu Photonics, www.hamamatsu.com) digital camera mounted on an Olympus BX51 microscope, and MetaMorph (Universal Imaging/Molecular Devices, www.moleculardevices.com/) imaging software.

## RESULTS

### The SSGP-11B1-encoding gene and related cDNAs

**Figure 1.**
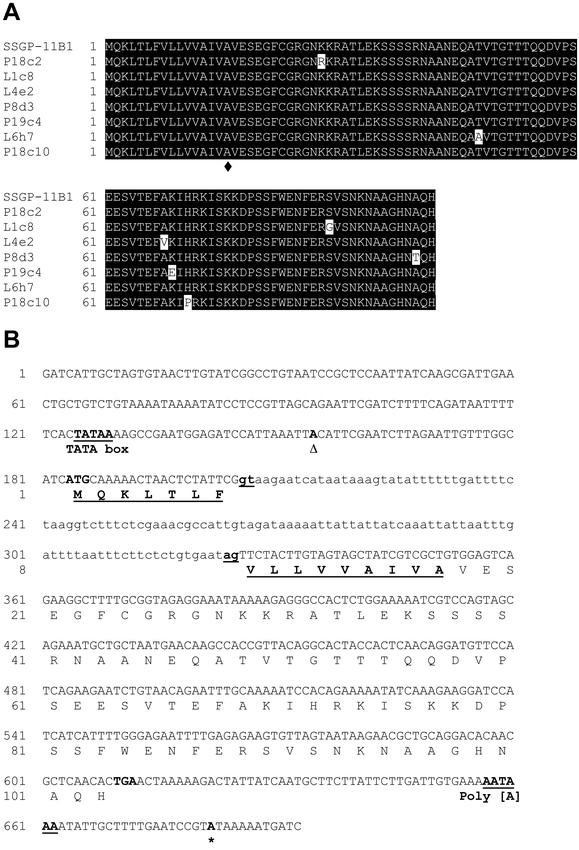
The SSGP-11B1-encoding gene and related cDNAs. A. Sequence alignment of putative proteins encoded by related cDNA clones. Amino acid differences are high-lighted by white residues. The symbol ♦ indicates the ending of putative secretion signal peptide. B: Nucleotide and predicted amino acid sequences of the SSGP-11B1-encoding gene. The sequence of the intron is shown in small letters. The putative TATA box, cDNA starting and ending positions, intron splicing donor and acceptor, and Poly (A) addition signal sequences are bold and underlined. The symbols Δ and * represent the starting and ending positions of the corresponding cDNA. Predicted amino acid sequence is shown under the nucleotide sequence. Putative secretion signal peptide of the protein is bold and underlined.

Eighteen full-length cDNA clones were identified that were related to the SSGP-11B1-encoding gene from three thousand clones that were randomly sequenced from a salivary gland cDNA library (Chen et al. 2004). Eleven of these cDNA clones are identical. The other seven cDNA clones contain minor (less than 2%) sequence variations. The proteins encoded by these seven cDNA clones are very similar to the SSGP-11B1 protein, the changes resulting in single amino acid substitutions (Fig. 1A).

An SSGP-11B1 cDNA clone was used to probe a BAC library (Liu et al. 2004) and six BAC clones were identified that cross-hybridized with the cDNA probe. The genomic fragment carrying the SSGP-11B1-encoding gene from one of the BAC clones was subcloned and sequenced. The nucleotide and predicted amino acid sequences of the SSGP-11B1 gene are shown in Fig. 1B. The 5′-noncoding region contains a putative TATA box. Sequence alignment between the gene and the corresponding cDNA revealed that this gene contains a small intron consisting of 120 base pairs in the coding region. A predicted poly(A) addition signal was observed in the 3′-noncoding region. The predicted protein contains 103 amino acids. The first 16 amino acids constitute a putative secretion signal peptide. The gene encodes a mature (predicted) protein of 9.5 kDa with an isoelectric point of 8.9.

### The SSGP-11C1 and SSGP-11C2-encoding genes, and related cDNAs

**Figure 2.**
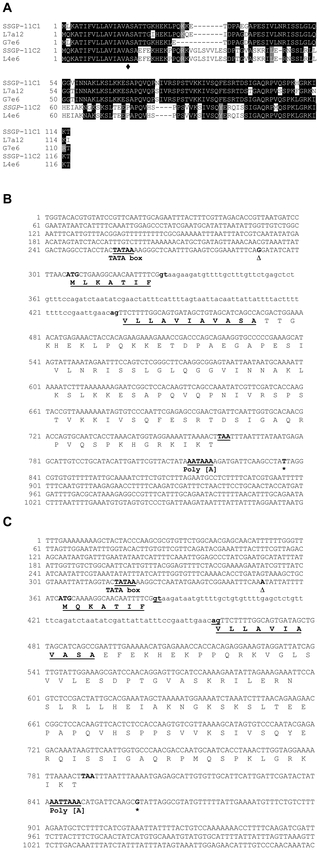
The SSGP-11C1- and SSGP-11C2-encoding genes and related cDNAs. A. Sequence alignment of putative proteins encoded by these two genes and related cDNA clones. B: Nucleotide and predicted amino acid sequences of the SSGP-11C1-encoding gene. C: Nucleotide and predicted amino acid sequences of the SSGP-11C2-encoding gene. The symbols were the same as described in Figure 1.

Five full-length cDNA clones were identified that were predicted to code for a group of proteins designated the SSGP-11C family. cDNAs encoding two of these proteins, designated SSGP-11C1 and SSGP-11C2, were approximately 75% identical in their coding regions. The other three cDNA clones encoded proteins that were similar to one or the other of these two proteins except for several amino acid substitutions and small insertions/deletions (indels) (Fig. 2A).

A genomic fragment corresponding to the SSGP-11C1-encoding cDNA was characterized from a BAC clone that hybridized to the cDNA probe. The nucleotide and predicted amino acid sequences of the SSGP-11C1-encoding gene are shown in Fig. 2B. The 5′-noncoding region contains a putative TATA box. Sequence alignment between the gene and the corresponding cDNA revealed that this gene contains a small intron consisting of 109 base pairs in the coding region, in a similar position to that of the small intron in the SSGP-11B1-encoding gene. A poly(A) addition signal was found in the 3′-noncoding region. The predicted protein contains 115 amino acids. The first 18 amino acids constitute a putative secretion signal peptide. The gene encodes a mature (predicted) protein of 10.5 kDa with an isoelectric point of 10.34.

Similarly, the nucleotide and predicted amino acid sequences of the SSGP-11C2-encoding gene are shown in Fig. 2C. There is a putative TATA box. The gene contains a small intron consisting of 74 base pairs in the same position as that of the small intron in the SSGP-11C1-encoding gene. The predicted protein contains 117 amino acids. The first 18 amino acids constitute a putative secretion signal peptide. The gene encodes a mature (predicted) protein of 11.0 kDa with an isoelectric point of 10.13.

### Structural conservation between the SSGP-11C1- and SSGP-11C2-encoding genes

The proteins encoded by the SSGP-11C1 and SSGP-11C2-encoding genes share structural similarity despite significant divergence. Except for one amino acid difference, the two proteins share an otherwise identical secretion signal peptide (Fig. 2A). Similarly, there is only one residue difference between the two proteins in the nineteen amino acids at the C-terminal. In addition, localized conservation can also be found in the diversified N-terminal portion of the mature protein.

**Figure 3.**
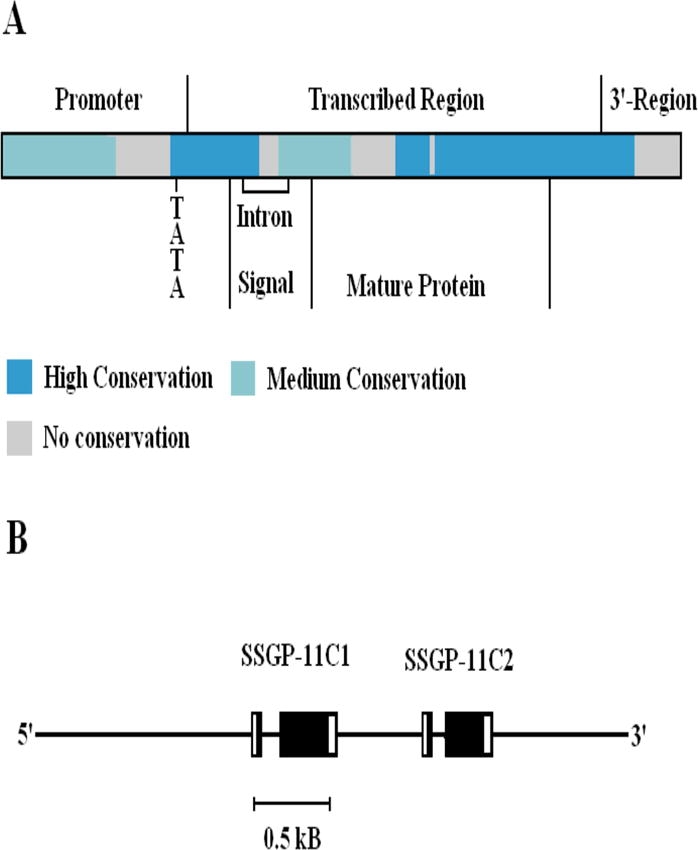
Genomic organization and sequence similarity between the SSGP-11C1 and SSGP-11C2-encoding genes. A: Sequence similarity. The sequence alignment was generated by the BLASTN program. The bars with different colors represent different levels of conservation between these two genes. The alignment is divided into different regions according to their functions, including promoter, transcribed region (regions coding for signal peptide and mature protein), and 3′-region. A single intron is within the region coding for the signal peptide. “TATA” indicates where the putative TATA box is located. B: Genomic organization. The boxed regions represent exons with the coding region in black.

At the nucleotide level, sequence comparison revealed that most parts of the SSGP-11C1 and SSGP-11C2-encoding genes are conserved (Fig. 3A). The four major regions that are not conserved are located immediately before the TATA box, within the intron, in the N-terminal region of the mature-protein-coding region, and in the 3′-untranscribed region, respectively.

Structural conservation between SSGP-11C1 and SSGP-11C2 indicated that the two genes arose by gene duplication. Consistent with this possibility, analysis of the genomic organization revealed that the SSGP-11C1 and SSGP-11C2-encoding genes are tandem repeats located within a 1.5 Kb DNA fragment (Fig. 3B).

### Structural conservation among gene families

**Figure 4.**
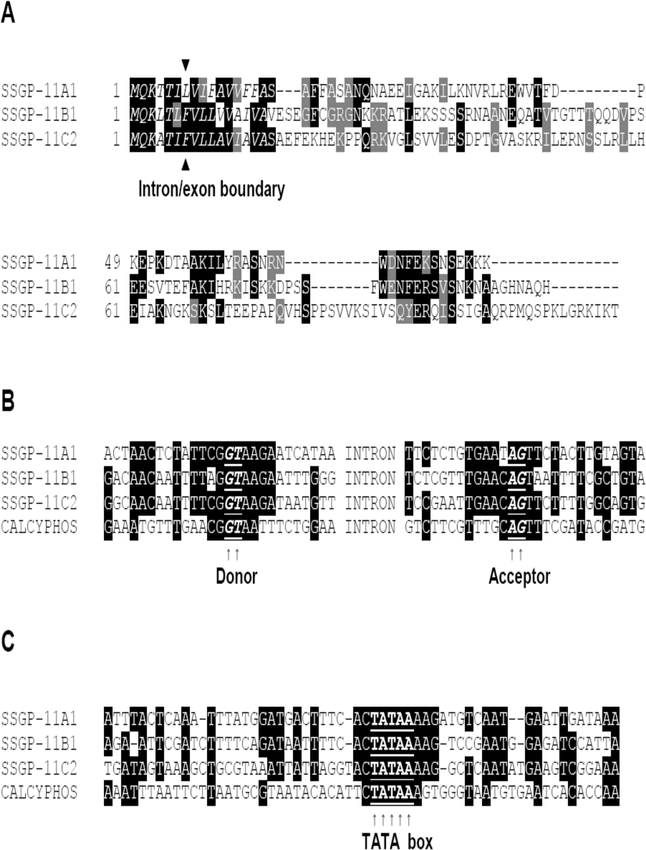
Structural similarity among the SSGP-11A1, SSGP-11B1, and SSGP-11C2-encoding genes. A: Amino acid sequence alignment. The first 16 amino acids (italic) constitute a putative secretion signal peptide. Black residues indicate identical amino acids between at least two proteins. Gray residues represent conserved substitutions. White residues represent amino acid differences. Dashed lines indicate absence of amino acids in specific proteins. The conserved location of the intron (between F and V) is indicated by the symbols ▴ and ▾. B: Conservation in nucleotide sequence at intron/exon splicing boundaries. The alignment was made using standard program (see Experimental Protocol section). CALCYPHO represents calcyphosine, an unrelated gene from the Hessian fly. Black areas represent residues identical at least 3 sequences. Intron splicing donor and acceptor are bold and underlined and indicated by arrows. C: Conservation in the promoter region. Putative “TATA” box is bold and underlined and indicated by arrows.

According to the genomic organization and structural conservation, SSGP-11C1, SSGP-11C2, and related cDNAs can be considered a gene family. SSGP-11B1 and the previously characterized SSGP-11A1 (Liu et al. 2004) represent two other families. Sequence analysis indicated that there was structural conservation among these gene families. First, the number, the location, and the phase of the intron are conserved. There is a single intron in all genes and the location of the intron is the same in terms of protein encoding (Fig. 4A). Second, the secretion signal peptide, which is different from those found in other SSGPs (Chen et al. 2004, Liu et al. 2004), is conserved among all three families (Fig. 4A). In addition to the conserved secretion signal peptide, there is also localized conservation in the mature protein among the three sequences (Fig. 4A).

At the nucleotide level, there are also conservations around the intron/exon boundaries (Fig. 4B) and in the putative promoter region (Fig. 4C). In the sequence alignment of the intron/exon boundaries with the three genes together with an unrelated Hessian fly gene encoding a calcyphosine-like protein, there are 29, 30, and 33 residues conserved in the SSGP-11A1, SSGP-11B1, and SSGP-11C2-encoding genes, respectively (Fig. 4B). In comparison, only 19 residues are conserved in the calcyphosine-encoding gene. Similar observations can also be found in the alignment of the putative promoter region. There are 29–30 residues conserved in all the SSGP genes, while only 18 residues are conserved in the calcyphosine gene (Fig. 4C). The structural conservation among these gene families suggests that they may have arisen from the same origin through gene duplications. The intron/exon boundaries and the promoter region of the duplicated genes diversified at a lower rate than the rest of the gene, which could be due to regulatory roles in RNA splicing and in gene expression.

### Gene expression analysis

**Figure 5.**
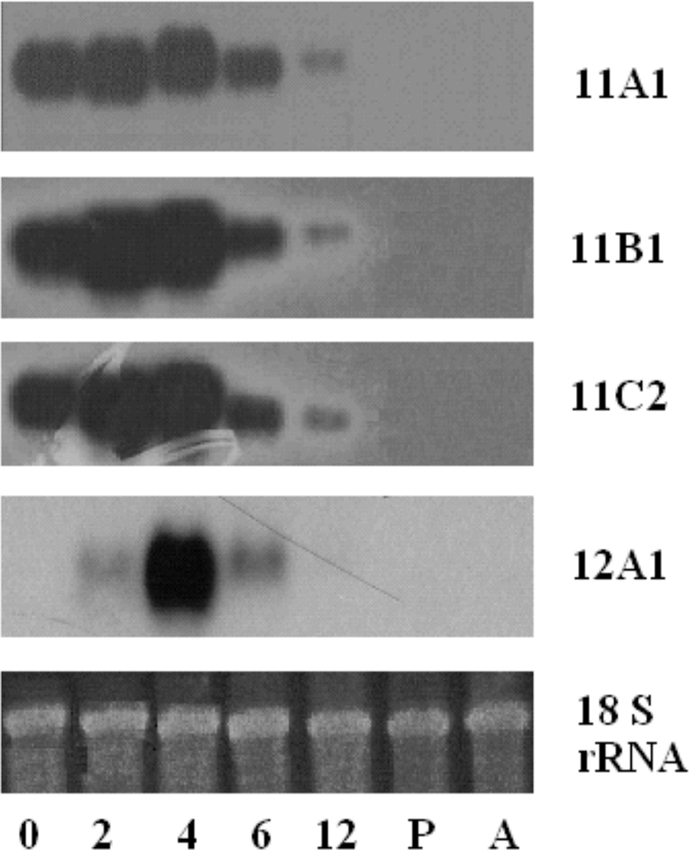
Northern blot analysis. The numbers 0, 2, 4, 6, and 12 underneath the figure represent samples extracted from 0-day (freshly hatched), 2-day, 4-day, 6-day, and 12-day old larvae, respectively, while the letters P and A represent samples extracted from pupae and adults. A blot was hybridized to a probe derived from the SSGP-11A1 gene (11A1). After the radioactivity stripped or decayed (no image by 48 hours exposure), the same blot was rehybridized sequentially to probes specific to SSGP-11B1 (11B1), SSGP-11C2, and SSGP-12A1, an unrelated SSGP gene (Liu et al. 2004). An image of the 18 S rRNA for the blot was included to show that the loading amount of RNA in each lane was about the same.

The conservation in the promoter region suggested that these gene families might have similar regulatory mechanisms controlling their expression. Northern blot analysis indeed demonstrated very similar or the same expression profiles among the SSGP-11A1, SSGP-11B1, and SSGP-11C2-encoding genes (Fig. 5). This was in comparison with a very different expression profile for a different gene, the SSGP-12A1-encoding gene, which was only transiently expressed in 2- to 6-day old larvae (Liu et al. 2004). All the SSGP-11-encoding genes were abundantly expressed in freshly hatched larvae and reached a maximum in 2- to 4-day old larvae. The RNA level was significantly lower in 6-day old larvae and became barely detectible in 12-day old larvae. No RNA could be detected in pupae and adults. The first 4 days are the first instar, which is the critical stage to determine if a larva lives or will die in compatible or incompatible interactions with wheat plants (Hatchett and Gallun 1970; Byers and Gallun 1971). The predominant expression of these genes in the first instar and all of the four genes peaked in 4-day old larvae suggested that they may play a critical role in Hessian fly virulence/avirulence.

### Southern blot analysis

**Figure 6.**
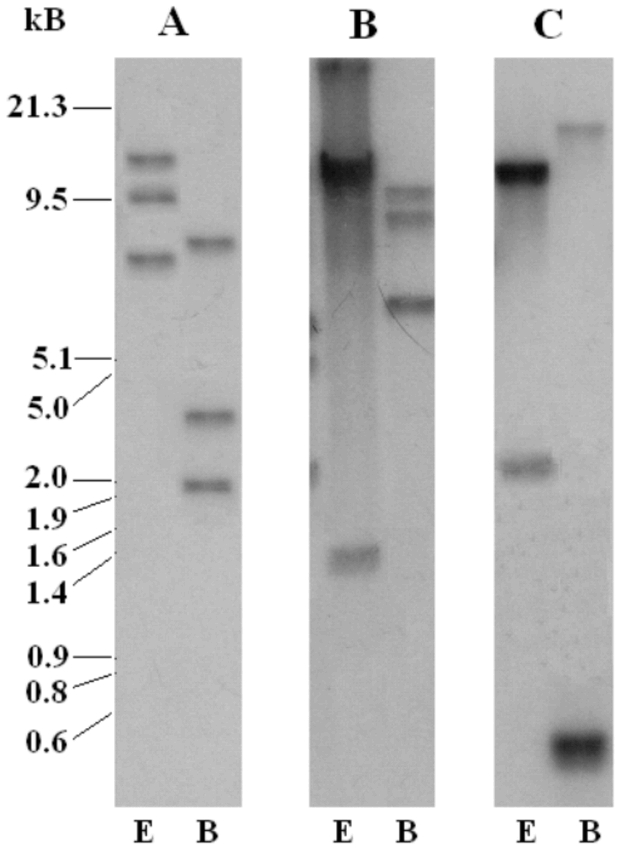
Southern blot analysis of genomic DNA. Genomic DNA was isolated from first instar larvae and digested with restriction enzymes EcoRI (E) or *Bam*HI (B). The blot was hybridized to the SSGP-11A1 cDNA (panel A). After the blot was stripped or decayed, the blot was rehybridized sequentially to probes specific to SSGP-11B1 (panel B) and SSGP-11C2 (panel C). DNA size markers were indicated on the left of the figure.

Southern blot analysis with genomic DNA isolated from Hessian fly larvae revealed restriction fragments with specific probes (Fig. 6). A probe specific to the SSGP-11A1-encoding gene detected three fragments with both *Eco*RI and *Bam* HI restriction enzymes (Fig. 6A). Since there are no *Eco* RI or *Bam* HI sites in the gene and the size of the gene is less than 1 kb (Liu et al. 2004), the three fragments indicated that there are at least three SSGP-11A1 related genes in the Hessian fly genome. Alternatively, there are allelic variations in the SSGP-11A1 locus that produce different restriction fragments on the Southern blots.

Multiple fragments were also detected in Southern blots with a probe specific to the SSGP-11B1-encoding gene (Fig. 6B). There is an *Eco* RI and a *Bam* HI site in the SSGP-11B1-encoding gene, but the *Eco* RI site is not within the region where the probe was generated. The single *Bam* HI site in the SSGP-11B1 probe should only detect two hybridization bands if there is only one gene. The multiple fragments with sizes greater than 1 kb (the SSGP-11B1-encoding gene is less than 1 kb) on Southern blots again indicated that there are multiple genes related to SSGP-11B1 in the Hessian fly genome, or that there are allelic variations in the SSGP-11B1 locus.

Two fragments were detected on Southern blots with a probe generated from the SSGP-11C2 encoding cDNA (Fig 6C). Since SSGP-11C2 has sequence similarity with SSGP-11C1, the probe may also hybridize to both subfamilies of genes. However, there are no *Eco* RI or *Bam* HI sites in the genomic fragment that carries both the SSGP-11C1 and SSGP-11C2-encoding genes. The two bands detected by the probe indicated that there are other related gene(s) in addition to the SSGP-11C1 and SSGP-11C2 encoding genes unless there are allelic variations in these two loci.

### Chromosomal localization

**Figure 7.**
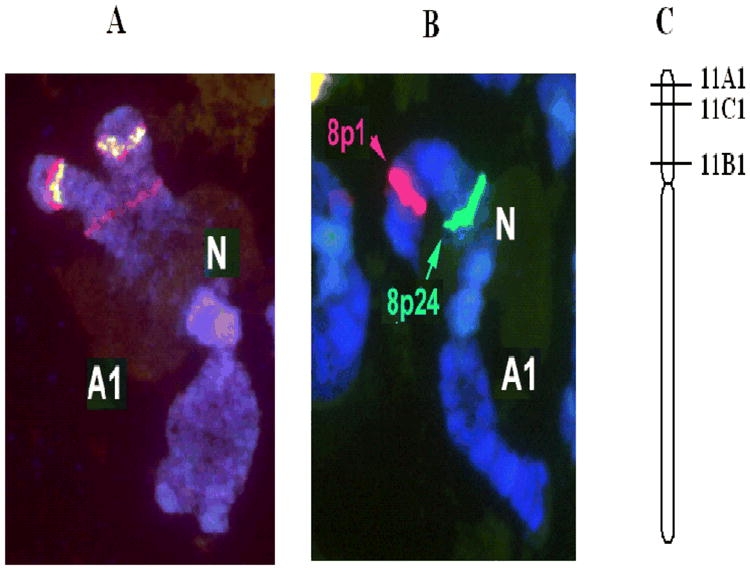
Fluorescent *in situ* hybridization (FISH) analysis. A: Relative positions of the SSGP-11A1 and SSGP-11C1 genes. BAC clones 3k23 (containing SSGP-11A1) and 8p1 (containing SSGP-11C1 and -11C2) were labeled with red and green fluorescent dyes, respectively, and hybridized to polytene chromosomes prepared from larval salivary glands. A yellow image was observed in the overlapping region, indicating that these two BACs were located next to each other. In addition to a strong image at the distal region of chromosome A1 with the 3k23 (red) probe, a weak image was also detected in the proximal region of the same chromosome. This weak image appeared to be in the same region where the SSGP-11B gene family (see panel B), and might be caused by related hybridization. B: Relative positions of the SSGP-11B1 and SSGP-11C1 genes. BAC clones 8p24 (containing SSGP-11B1) and 8p1 (containing SSGP-11C1 and -11C2) were labeled with green and red fluorescent dyes, respectively, and hybridized to polytene chromosomes prepared from larval salivary glands. A single location for each BAC probe was observed in the Hessian fly genome. C: A physical map of the three SSGP genes. A map was constructed to show the relative chromosomal localization of the SSGP-11A1, SSGP-11B1, and SSGP-11C1 (and -11C2) genes according to the FISH analysis.

Fluorescence *in situ* hybridization (FISH) was performed to determine the relative physical locations of the three gene families using a two color system. Figure 7A shows the result of FISH using BAC clones 3k23 (containing SSGP-11A1) and 8p1 (containing SSGP-11C1 and SSGP-11C2), while Figure 7B is the image of FISH using BAC clones 8p1 and 8p24 (containing SSGP-11B1). According to the FISH results, SSGP-11A1 and SSGP-11C1 are in the distal region of the short arm of chromosome A1. SSGP-11B1 is in the proximal region of the same chromosome arm (Fig 7C).

## DISCUSSION

The SSGP-11C1 and SSGP-11C2 encoding genes share relatively high sequence similarity and are located in a 1.5 kb DNA fragment as tandem repeats. This suggests that they originated from a gene duplication event followed by sequence diversification. The conservation in most of the mature protein (Fig. 2A) indicates functional similarity between these two proteins. The above evidence, suggests that SSGP-11C1 and SSGP-11C2 are two members of a gene family, the SSGP-11C family. On the other hand, the high degree of sequence diversification between the SSGP-11C family and the SSGP-11A1 and SSGP-11B1-encoding genes indicated they are distinct gene families that are likely to perform different functions, or possibly have different specificities.

Although the DNA sequences have little noticeable sequence similarity, and very little homology exists in the predicted amino acid sequences (data not shown), structural conservation among members from the three families can be found. For example, a single intron was found in all gene families (Figs. 1 and 2, Liu et al. 2004) and the location of the intron was exactly the same, namely in the eighth codon of the putative signal peptide (Fig. 3B and Fig. 4A). Not only the position was the same, the phase of the intron was also identical in all these genes. It is unlikely that the same position and identical phase of the intron was achieved through convergence of genes of different origin. In fact, different numbers of introns were found in different locations and in different phases in other SSGP-encoding genes (Liu et al. 2004; Chen et al., unpublished) regardless of their expression patterns or protein structures. The conservation in the number and the location of the intron also suggests that members from the three gene families arose from a common origin through gene duplications.

Consistent with this hypothesis, localized conservation exists among these genes at the nucleotide and amino acid levels. At the amino acid level, the secretion signal peptide and a short region at the C-terminal are relatively conserved (Fig. 4A). Such conservation could not be found when comparing these proteins with other unrelated SSGPs (data not shown). At the nucleotide level, conservations can also be found in the intron/exon boundaries (Fig. 4B) and in the promoter region (Fig. 4C) in addition to the signal peptide coding region. The localized conservations were consistent with the possibility that the signal peptide coding region, the intron/exon boundaries, and the promoter region diversified at a slower rate after gene duplication because of a functional requirement for secretion, RNA splicing, and expression regulation. The other parts of the genes diversified at a higher rate to gain new functions for the proteins. The conservation in the promoter region could explain that the expression profiles were essentially the same for all the three families, and that they were very different from those of other unrelated SSGP genes (Chen et al. 2004; Liu et al. 2004).

Based on above evidence we conclude that SSGP-11C1 and SSGP-11C2 are two members of a gene family, the SSGP-11C family. Each gene family appeared to have multiple members according to genomic Southern blot analysis. In addition, there were multiple cDNAs that encoded proteins with sequence variations in each gene family. Even though some of the cDNA variations could be caused by polymorphisms, many of them were likely to represent paralogs because of the predicted amino acid divergence and the presence of relatively large indels (Fig. 2B).

The two genes (SSGP-11C1 and SSGP-11C2) in the SSGP-11C family are tandem repeats located within a 1.5 Kb DNA fragment (Fig. 3B). Evidence also indicated that multiple members in other families were also clustered within a short chromosome region. For example, Southern blot analysis of a 145 Kb BAC clone produced the same hybridization pattern (data not shown) revealed by genomic Southern blot analysis, indicating that multiple genes from the SSGP-11B family were contained within this BAC clone. In addition to clustered family members, the SSGP-11A and SSGP-11C encoding gene families are also located next to each other on the distal region of the short arm of chromosome 1A. The localization of the SSGP-11B family in the proximal region of the same chromosome arm might be caused by a chromosome rearrangement.

It is not clear at present what kind of function this group of proteins may perform. Sequence comparison using various programs detected no significant homology with genes or proteins in known databases including the GenBank. To elucidate the exact biological functions of these genes, we are currently generating recombinant proteins and producing antibodies. Our future direction is to determine where these proteins are located once they are injected into plants and which proteins from host plants they are interacting with.
